# Neuroethics and fMRI: Mapping a Fledgling Relationship

**DOI:** 10.1371/journal.pone.0018537

**Published:** 2011-04-22

**Authors:** Alex Garnett, Louise Whiteley, Heather Piwowar, Edie Rasmussen, Judy Illes

**Affiliations:** 1 National Core for Neuroethics, University of British Columbia, Vancouver, British Columbia, Canada; 2 School of Library, Archival, and Information Studies, University of British Columbia, Vancouver, British Columbia, Canada; 3 National Evolutionary Synthesis Center, Durham, North Carolina, United States of America; French National Centre for Scientific Research, France

## Abstract

Human functional magnetic resonance imaging (fMRI) informs the understanding of the neural basis of mental function and is a key domain of ethical enquiry. It raises questions about the practice and implications of research, and reflexively informs ethics through the empirical investigation of moral judgments. It is at the centre of debate surrounding the importance of neuroscience findings for concepts such as personhood and free will, and the extent of their practical consequences. Here, we map the landscape of fMRI and neuroethics, using citation analysis to uncover salient topics. We find that this landscape is sparsely populated: despite previous calls for debate, there are few articles that discuss both fMRI and ethical, legal, or social implications (ELSI), and even fewer direct citations between the two literatures. Recognizing that practical barriers exist to integrating ELSI discussion into the research literature, we argue nonetheless that the ethical challenges of fMRI, and controversy over its conceptual and practical implications, make this essential.

## Introduction

Functional magnetic resonance imaging (fMRI) is an increasingly popular noninvasive method for studying the functional anatomy of the human brain. fMRI experiments correlate mental phenomena with a hemodynamic measure of brain metabolism, and the resulting data are often presented as an anatomical map of the brain regions purportedly involved in the mental phenomenon of interest. The domains in which fMRI has been applied are highly diverse, ranging from language comprehension or short-term memory to personality traits, political behavior, or aesthetic judgment [1]. fMRI raises key ethics questions throughout the research process: from the conceptualization of experiments through their design, conduct, and analysis, to the interpretation and dissemination of results, and the possible implications and applications of research [2]–[4]. Here, we briefly review the neuroethics of fMRI [5], focusing on issues most relevant to the researcher. We consider arguments for bringing discussion of ethical, legal, and social implications (ELSI) into the primary research literature, and review barriers to such activity. We then use bibliometric methods to map out the ELSI landscape for fMRI, highlight salient topics and citation relationships, and reveal the degree to which calls for ethics discussion in the research literature have been heeded.

In the research context, fMRI raises familiar issues of informed consent [6] and hotly debated questions about the investigation of incidental findings [7], inflected by the potential impact of neurological conditions on cognition and selfhood [5], [8]. In the clinical context, explaining and treating mental illness in biological terms is deeply controversial, and it remains unclear whether neuroimaging practices for diagnosis and treatment will benefit patients [9]. The effect on participants of taking part in neuroimaging research studies of psychiatric conditions is also unclear; recent studies suggest that brain scans are powerful tools for shaping or reinforcing concepts of a ‘disordered’ self [10]–[11]. fMRI also has potential applications in legal, educational, and economic contexts, ranging from lie detection to the justification of cognitive enhancing drugs in educational settings [12]–[15]. Although many of these applications are currently speculative, and while there is a danger of exaggerating the real-world consequences of neuroscientific findings [16], the impact of fMRI research on ways of thinking about education, psychiatric classification, economics, and responsibility is already substantial [17]–[22]. In the philosophical context, imaging studies of human judgment, emotion, personhood and responsibility all have controversial implications for ethical theory [23]–[25]. These debates are intensified by the question of how brain images should be communicated: fMRI scans are highly processed representations of an indirect measure of neural activity, but are often described as if they are direct snapshots of the mind in action. They are thus rhetorically powerful, and their apparent directness can obscure a range of contingent interpretations and underlying conceptual commitments [26]–[27]. Take, for example, the presentation of two scan images, one from a group of patients with a particular psychiatric diagnosis and one from a control group: the scans can imply both that the categories are distinct and that they are biologically-based while neither may be the case [28].

Many of these issues are directly relevant to the fMRI researcher [29]. In addition to the more transparent ethics issues surrounding the conduct of research, the conceptualization and dissemination of research raises difficult questions about the researcher’s expertise and public responsibility. In designing controlled laboratory studies of complex concepts such as anger, wisdom, or empathy, there is much to be learned from philosophical literatures and studies in the social sciences about the implications of operationalizing these concepts in particular ways [16]. The movement of fMRI research into media reports and real-world applications is also deeply entangled with the researcher’s activities, contra assertions that research is neutral to whatever might be made of it. The nascent field of neuroethics aims to highlight, inform, and address underexplored ethical, legal, and social issues arising from neuroscience, and to encourage discussion among researchers and research communities [30]–[33]. Here we ask whether neuroethics has been successful in penetrating the neuroimaging research domain by examining citation patterns. Investigating bibliometric trends such as citation patterns is a key method for understanding the development of nascent fields such as neuroethics and their interaction with existing disciplines, and for tracking the evolution of their often mutable terminologies [34].

One might predict that primary research articles would *not* discuss (neuro)ethics in depth, with researchers assuming that institutional ethics approval is sufficient, and having little incentive to engage further [35]–[36]. This is of concern, as ethics training and approval is often superficial and inadequate to the particular quandaries of neuroimaging [5]. However, one might also expect that the increasing degree of overlap between the potential real-world implications of fMRI research and its subject of study – for example, in experiments pertaining to human nature, mental contents, and moral reasoning – would be reflected in increased overlap in fMRI and ethics content, and in cross-citations between the two literatures. In addition, there have already been calls for researchers to engage more deeply in ethical, social, and legal debate within science journals [33], [37], neuroethics topics are increasingly discussed in the popular press [38], [39], and neuroimagers are increasingly called upon to defend their encroachment on the traditional domains of the humanities [40], [41].

## Methods

For bibliometric methods to accurately describe the shape of a particular field, it is crucial that search strategies, inclusion criteria, and visualization practices are appropriate to the domain [42]. We therefore developed the methodological procedure described below through collaboration between experts in information science, scientific publication, neuroimaging, and neuroethics. Full details are included in Table 1 and in the Appendix S1.

**Table 1:** Research Questions and Data Sets.

Research Questions	Data Set
Is discussion of the ethics issues surrounding fMRI taking place, and if is it described using the term “neuroethics”?	Academic papers containing permutations of the phrases “functional magnetic resonance imaging,” “ethics,” “neuroethics” Source: PubMed.
What are the salient topics among articles discussing both fMRI research and ethical, legal, and social issues?	Academic papers containing permutations of the phrases “functional magnetic resonance imaging,” “ethics,” “legal” (see Appendix S1). Source: ISI Web of Science.
Are fMRI articles, and those discussing ethical, legal, and social issues, citing each other?	Original fMRI research articles, based on detailed machine-derived query and manual curation. Source: PubMed.ELSI research articles, based on detailed query. Source: Scopus.

### 1. Tracking Overlapping fMRI and Neuroethics Publications

We took as a starting point Seixas and Basto's (2008) bibliometrics analysis of neuroethics, the only other study of this nature to date [43]. Although Seixas and Basto's (2008) focus was on impact factor and nation of origin, and on issues affecting radiologists [43], we revisited their coding guide to provide continuity for the present content analysis. We conducted searches for all articles indexed in PubMed containing permutations of the phrases “functional magnetic resonance imaging,” and “ethics,” as well as all literature containing the term “neuroethics” specifically, and recorded publication numbers for each year (see Appendix A in Appendix S1).

### 2. Mapping the Intersection of fMRI and ELSI

We used an ISI Web of Science query to identify scholarly articles published between 1999 and 2009 that contain both “fMRI” and “ethical” or “legal” as topic terms (see Appendix B in Appendix S1).We discarded all articles that did not have abstracts available and ‘false hits’ (articles in which the mention of ELSI was non-substantive, e.g., a cursory reference without any discussion within the text, or referred to a standard ethics approval process). We sorted the remaining articles according to nominal topic categories, augmenting the scheme used by Seixas and Basto (2008) to reflect new facets of the literature [43]. Appendix C in Appendix S1 lists the categories in the original and augmented coding scheme – the only two independent additions were a category including research on the neurobiology of moral and ethical judgments, and a meta-ethics category to encompass papers and reviews considering neuroethics itself.

A final coding scheme was applied by two authors (LW and AG), and the minimal number of disagreements were resolved through discussion. Since this kind of coding is inevitably shaped by pre-existing ways of conceptualizing the literature, both in the design and application of the codes, we also conducted an automated analysis to reveal clusters of co-citing papers. This provides a more data-driven picture of ELSI topics surrounding fMRI, weighted by a richer measure of the prominence of each paper in the citation landscape. We submitted the original query results to the CiteSpace II platform for visualizing patterns in Scientific Literature, which generates an directed graph showing the citation links (edges) between papers (nodes, each representing a single published article) [44]. Less well-connected papers were filtered out using a standard pruning procedure to aid visualization. This procedure is described in detail in Appendix D in Appendix S1.

We also used the CiteSpace II platform to apply machine-derived labels to each cluster, constructed from noun phrases appearing in the constituent article titles, abstracts, and indexing terms. Such machine-derived labels are seldom a close approximation of natural scientific language, and we therefore translated the machine-derived labels into more salient terms already utilized in the neuroethics literature. This translation process was evaluated by a team with expert knowledge of the domain.

### 3. Citation Analysis

To specifically investigate direct citations between articles from the two domains, we constructed a PubMed query to identify original research articles that used fMRI with human subjects between 1999 and 2009. We excluded review articles, editorials, meta-analyses and commentaries. A detailed analysis of how we constructed this query is provided in Appendix B in Appendix S1.

For the purpose of data management, we retained a randomized subset of up to 200 articles for each year and indexed the articles by their unique PubMed identifier. To obtain metadata on the articles, the articles they referenced, and those in whose reference lists they appeared, we submitted the PubMed identifiers to the Scopus citation database. For both the cited and citing articles, we then identified the subset that contained ELSI content using three filtering criteria. The criteria were developed through both automated and manual analysis of the accuracy and exhaustiveness of their results. At least one of three criteria had to be met for an article to be included (details are provided in Appendix E in Appendix S1):

- journal title contained the words or word fragments “ethic,” “poli-”, “philosophy,” “law;” or match a list of ISSNs (for journals with less keyword-salient titles),
- article title contained the phrase “informed consent, ” or the article title or abstract contain the words or word fragments, “ethic,” “justice,” “stigmati-,” “responsibil-”, or “personhood”.
- PubMed identifier of the article listed in Scopus and identified by the PubMed “bioethics[sb]” filter.

As a final check, we manually reviewed all article titles and abstracts identified by this filtering process and eliminated from further analysis those not considered by the authors to be an article about ELSI issues. We also eliminated citations not considered to reflect substantive discussion of research ethics or ethical, legal, and social implications. This most often occurred when a) a paper in the ELSI literature was cited for a background statistic not related to research ethics or implications, or b) when ELSI papers were cited with respect to the content of the fMRI study – in other words, cited by studies using neuroimaging to study moral, legal, or ethical decision-making. The exclusion of citations referencing these moral correlate studies allowed us to more accurately map citations by fMRI research articles that indicate an additional and substantive engagement in questions arising from the ethics of research or its implications. Note that where a paper with a moral correlates citation *also* cited an ELSI paper in the context of discussion of research ethics or implications the latter citation was retained. To determine the context of ELSI discussion, we located and retained the paragraphs in which citations occurred.

## Results

### 1. fMRI and Neuroethics Publications

**Figure 1:**
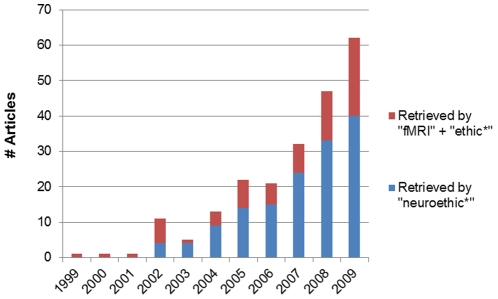
Prevalence of neuroethics publications, 1999–2009.

As illustrated in Figure 1, the prevalence of neuroethics publications has increased by an average of 39% each year since the term was formalized in 2002 [31], but this number is small overall, with 40 publications including the term “neuroethics” in their title or abstract in 2009. However, this does not include those articles that discuss both fMRI and ethics without using the term “neuroethics”. Increases in the number of publications considering ethics issues associated with fMRI has risen in turn with those containing an eponymous mention of neuroethics at an average of 51% more publications each year.

### 2. Landscape of Intersecting fMRI and Ethics Content

**Figure 2:**
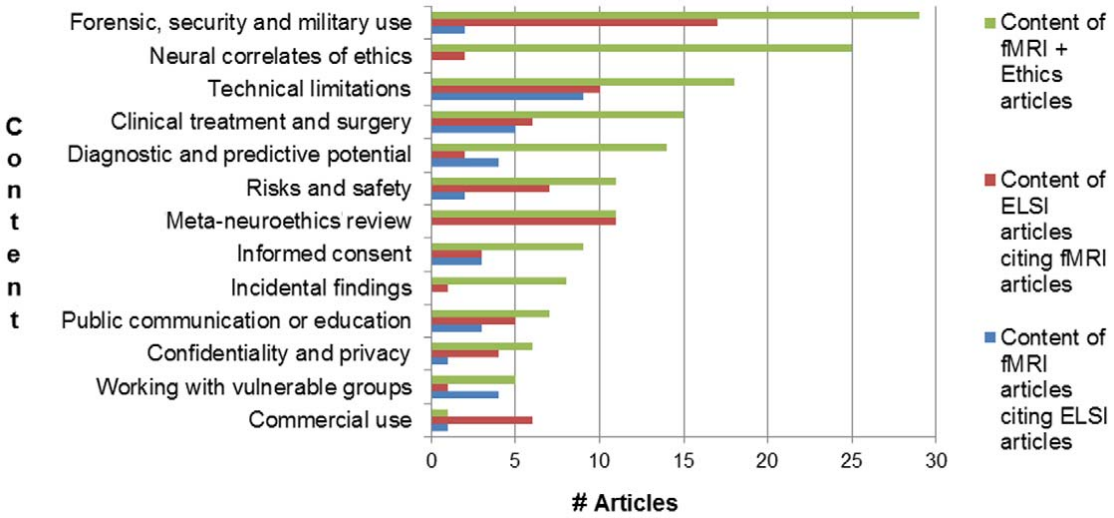
Content analysis of neuroethics publications and citation contexts.

Of the 134 articles referring to both fMRI and ELSI issues returned by our query, 50 were eliminated for lack of substantial discussion of research ethics. The 84 remaining articles came from a broad range of journals across bioethics, the social sciences, and law. As illustrated in Figure 2, we observed extensive discussion of the forensic, security, and military use of fMRI, frequently in the context of neural correlates associated with guilty feelings or incriminating mental contents of people accused of crimes. In terms of ‘real-world’ implications, this focus on forensic, security, and military use was in marked contrast to the lack of discussion about commercial use, which is arguably the most pressing source of ethical concerns outside the research and clinical context – private imaging clinics already offer scans for lie detection and diagnosis of mental illness [45]. Articles that detailed the extension of neuroscience into legal and other domains often also discussed more general technical problems with generalizing fMRI results across individual human subjects, and from abstract laboratory tasks to real-world contexts. Among the articles discussing both fMRI and ethics, and among ELSI articles citing fMRI articles, there were fewer instances of citations relating to the discussion of classic research ethics issues such as informed consent, incidental findings, working with vulnerable populations, and confidentiality than relating to the implications and limitations of neuroimaging research. Among the fMRI articles themselves, citations of ELSI articles were more evenly distributed among these categories, though the small numbers involved limit the conclusions that can be drawn. It is also of interest that among the fMRI articles citing ELSI articles, the most common context was discussion of technical limitations, concordant with previous observations about the focus of critique in the print media [46].

**Figure 3:**
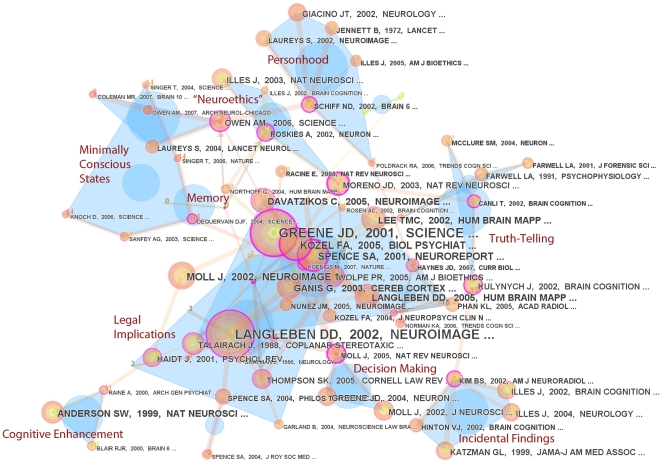
Citation network of neuroethics publications with topic clustering.

To provide a more data-driven complement to our qualitative topic analysis, we input the original 134 articles returned by our query into the CiteSpace II platform. Figure 3 illustrates the network resulting for the top cited articles. Each node represents a paper, and the size of the node and of the corresponding label reflects the number of times the paper was cited. The warmth of the node colour indicates the recency of citation: pink indicates the most recent, blue the oldest citations. The distance between the nodes indicates relatedness: if two nodes are close together, the papers they represent share similar content. Clusters of closely-related papers are indicated by free-form blue shapes. Their citation centre of mass is shown by the blue circle. The salient neuroethics terms used to translate the machine-derived labels for each cluster were: decision making, cognitive enhancement, personhood, incidental findings, legal implications, minimally conscious states, truth telling, and the term “neuroethics” itself. It is important to note that the 77 nodes in this network do not correspond exclusively to articles from the 134 articles originally extracted from the ISI database. Article nodes were incorporated based on a high degree of relatedness to the topic clusters, including any highly cited articles not part of the original data set. The only such article on our graph is the 1972 Lancet article in the personhood cluster.

The large mass in the center describes research into moral correlates, including fMRI studies of personhood, disgust, and racial bias. While this largest cluster contains at least one citation link out to every other cluster on our graph, it overlaps most heavily with articles considering legal implications. This relatedness between the neuroscience of ethics and its applicability to the law was thus observed in both our manual coding and the cluster analysis, and reflects the presence of discussion about the legal implications of fMRI in both the popular media and legal press [39], [46]–[47].

Articles about incidental findings and cognitive enhancement form relatively cloistered clusters. The most highly-cited and interconnected node in each cluster represents one of the earliest papers in the respective domain: respectively, Kim et al. (2002), discussing incidental findings in pediatric MR imaging [48], and Anderson et al. (1999) discussing the implications of early prefrontal cortex damage [49]. The connection between prefrontal cortex damage and cognitive enhancement may not be immediately obvious: brain injury in fact provides a striking context in which interventions are made to enhance cognitive or behavioral ability [50].

An eponymous neuroethics cluster also emerged, describing those articles judged most similar purely for their use of the term, and often arising in the context of early commentaries on the state and aims of the discipline [e.g., 51]. Overlapping with this cluster were those labelled “personhood” and “minimally conscious states”, suggesting that these domains are most closely associated with the neuroethics literature; future work could investigate the chronology of this interaction. Topics such as the diagnostic and predictive potential of fMRI are more pervasive and less effectively clustered, perhaps reflecting their methodological generality and reminding us that ELSI issues associated with fMRI span technical, research, academic, and applied domains.

### 3. Citation Analysis

#### 3.1 Citations of the Ethics literature

To explore the degree of co-citation between the fMRI and ELSI literatures, we retrieved 3,484 fMRI articles published between 1999 and 2009. The automated ethics filter selected an initial subset of 244 ELSI articles cited by the fMRI articles (see Section 2.3; Figure 2). Of these, we manually identified 137 as research articles investigating the neurobiology of moral or ethical behavior, leaving 107 articles concerned with fMRI research ethics or implications. We examined the citation context of the remaining 107 articles, yielding only 18 citations that took place as part of a substantive ethics discussion in an fMRI article. However, this very small proportion does appear to be increasing from a baseline of virtually zero, at an average rate of 22% per year over the last ten years.

The quotes below are illustrative examples of the citation contexts in which fMRI articles invoked ELSI articles as part of a substantial ethics discussion:

*“If lawyers and ethicists continue to debate whether lesion patients or psychiatric patients with functional deficits should be considered culpable for their immoral actions* [50], *it will be helpful to acknowledge that some brain regions might be involved in only specific subsets of moral processing because patients could conceivably be held culpable for some types of immoral actions but not for others. Although there is still much to explore, the data reported here lay the groundwork for many future interdisciplinary investigations”* [52].

*“This has led to concerns that some [minimally conscious patients], who retain an awareness of self or environment, are being ‘warehoused’, without adequate access to appropriate assessment or rehabilitation”* [53].

*“Even during this period of expanded application, examiners have cautioned that appropriate use of fMRI in special samples will require further investigation of the influence of cerebrovascular changes on the fMRI signal”* [54].

#### 3.2 Citations of the fMRI literature

Of the articles citing the 3,484 fMRI articles selected, 43 articles published across varied disciplines such as law or business ethics included a substantial discussion of research ethics or ELSI implications meeting our criteria. These increased over time at a greater rate than the citations of ELSI literature in fMRI, at an average rate of 36% per year over the last ten years. Many of the citations of fMRI articles in the ELSI literature occurred in the context of discussions about the technical limitations, persuasive power, or evidentiary constraints of neuroimaging. For example:

*“Neuroimaging data seem particularly compelling to lay people and may present a “unique danger because of the appearance of scientific neutrality”* [55].

*“Ethical issues related to rights against self-incrimination have caused some companies developing forensic neurotechnologies to claim that their products will only be used to exonerate the innocent*. *In this context, the false-negative problem creates a real dilemma for companies developing memory detection tools”* [56].

The ELSI literature also included several citations of fMRI studies of moral correlates, which were excluded from this analysis in order to reveal cross-citations indicative of substantive discussion of the ethics and implications of research itself. For example, Salvador and Folger [57] note that “*in an fMRI study, Robertson et al*. [*58*] *presented Executive MBA students with story segments, some of which had moral content, while others had none”,* in the context of a separate discussion of research ethics surrounding fMRI.

## Discussion

We began by arguing that that fMRI raises substantive ELSI issues for the conduct, application, and implications of research. We further argued that fMRI researchers should engage in debate about these ethical issues and the implications of research, and hypothesized that the increasing popularity and media coverage of neuroimaging studies, and discourse surrounding real-world implications and early commercial applications, might have led to growing cross-citation of the two literatures in recent years. Between 1999 and 2009 there have indeed been an increasing number of articles published with overlapping fMRI and ethics content. However, this number is surprisingly small in total, and we found very few citations of fMRI research by ELSI articles, and an even smaller number of fMRI articles that substantively cited the ELSI literature.

In many fMRI studies, the only acknowledgment of research ethics was a mention of informed consent – necessary, in many cases, for publication, and a frustrating confound in our attempts to identify more substantive discussion. For example, there were frequent citations of the World Medical Association (Declaration of Helsinki) ethical principles for medical research including human subjects. While review boards can ensure that research is being carried out in accordance with stated principles, they cannot replace a meaningful discussion of ethics, particularly with respect to unique and underexplored features of a new domain [5].

In addition to the reliance on ethics review boards, there are practical, institutional, and incentive barriers to scientists taking ownership of the ethics and implications of their research. Indeed, a survey recently conducted with neuroimagers in Canada [59] suggested that many neuroscientists are indeed concerned with ethics issues, but are unsure of a productive vehicle for discussion and communication. It is possible that more substantial ethics discussion takes place in reviews, policy articles, or grant applications, and future studies could investigate these additional domains. In any case, given the rich discussion within neuroethics, policy debate, and the popular press, the relative lack of ELSI content in the primary literature is somewhat surprising and deserves further investigation.

The central topic cluster in our graphical analysis included both moral correlates research, and discussion of the ethics issues associated with using fMRI to putatively reveal facets or contents of the mind – often referred to as mind-reading. In the direct citation analysis we excluded moral correlates research, but several articles included discussion of the neural basis of morality among other ethics issues. We argue that these two domains naturally inform each other: for example, neuroimaging research on phenomena relating to disgust and their possible implications for jury behaviour ostensibly addresses a question about the neural basis of an ethical phenomenon, but does not answer the question of how such knowledge ought to be used [24]. Several scholars have argued that the nature of bioethical enquiry precludes the kind of binary fact-value distinction implied by a separation between research ethics and the neural correlates of ethics [24], [60], and we suggest that an emphasis on such distinctions may stymie further debate.

Bibliometrics rests on the assumption that authors cite the most appropriate available references, that their literature review is exhaustive, and that chosen citations are objectively valid for the purpose intended. Beyond this assumption, “authors are free to do whatever they need to the earlier literature to render it as helpful as possible for their own arguments” [61]. Citation analysis does not carry any positive or negative polarity, and should not be taken to provide a measure of the quality of a given work. What is actually being tracked is indirect influence, along with a work's visibility or acceptance among a described community. This is invaluable to the descriptive analysis of the role of terminology in shaping the dissemination and structure of new research fields [62]. Indeed, in tracing the network of research that has shaped neuroethics, we have found threads weaving together from diverse literatures in sociology, anthropology, cognitive psychology, behavioural psychology, marketing, law, and others. Lest we claim to have rediscovered what was not lost, we note that these links often originate in deliberate attempts by researchers working to unite the disciplines. Here, we aim to contextualize these individual links by providing a broader picture of the cross-disciplinary locus of neuroimaging research and the academic discourses surrounding its ethical, legal, and social implications.

Safire's call for commonly defined terms to galvanize the development of neuroethics [31] has been only partially heeded – discussion of neuroethics issues is often not referred to as such. Following Seixas and Basto [43] we therefore investigated overlapping ethics and fMRI content, and found that this landscape reflected a wide variety of concerns discussed in the eponymous neuroethics literature, ranging from clinical practice to philosophical enquiry. In fluid and diverse fields, using bibliometric analysis can thus support the spread of research, concepts, and methodologies and reduce the degree of repetition and redundancy. Here, we suggest better dissemination of neuroethics literature and terminology, and the related and overlapping work that lies outside this terminological terrain, to help galvanize ethical discussion among fMRI researchers. Ethics means many different things to many different people, and citation analysis serves as a reminder that research is driven by a focus on substantive questions that cross disciplinary boundaries.

Through mapping citation patterns we have presented the most complete picture yet of the extension of ethics into published fMRI research, and the locus of fMRI in ethical, legal, and social commentary. We conclude by emphasizing the benefit of devoting at least some space in fMRI research articles to specific research ethics questions, and to discussion of the wider meaning, concrete implications, and conceptual underpinnings of neuroimaging findings. Situating such discussion in the context in which it is originally reported [63] has the potential to improve the dissemination and prominence of ethical discussion surrounding this potent technology, to ground debate about the nature and reach of its real-world implications, and to challenge assumptions of neutrality at every stage of the research process.

## Supporting Information

Appendix S1 (DOC)
