# Supplementary material for: Neuroethics and fMRI: Mapping a Fledgling Relationship
Source: PLoS One. 2011 Apr 22;6(4):e18537. doi: 10.1371/journal.pone.0018537 (PMC3081297; doi:10.1371/journal.pone.0018537)
Supplement: Appendix S1 — (DOC) [file pone.0018537.s001.doc]

**Appendix A. ISI Web of Science Query Construction**

Our initial query used to retrieve all articles containing both fMRI and ethics content, irrespective of a direction mention of neuroethics, is as follows:

Topic=("functional mr" OR "functional magnetic resonance" OR fmri)

AND (*ethic* or legal)

**Appendix B. PubMed Query Construction**

The query used to retrieve ELSI-relevant source journals is as follows:

*ethic*, poli*, philosophy, law, OR

ISSN matching "00930334" (Hastings Center Report), "0020174X" (Inquiry), "09515089" (Philosophical Psychology), "01638548" (Human Studies), "13557858" (Ethnicity and Health), "10158146" (Genetic Counseling), "08989621" (Accountability in Research), "10790969" (Health and Human Rights)

The query used to retrieve original (i.e. non-review) human subjects fMRI research articles from PubMed is as follows:

humans[mesh] AND "magnetic resonance imaging"[mesh]

AND (fmri[Title/Abstract] OR "Functional MRI"[Title/Abstract]

OR "Functional magnetic resonance imaging"[Title/Abstract]

OR "Functional MR Imaging"[Title/Abstract])

NOT "magnetic resonance imaging/ethics"[mesh]

AND Journal Article[ptyp] AND English[lang]

NOT (Editorial[ptyp] OR Letter[ptyp] OR Meta-Analysis[ptyp]

OR Practice Guideline[ptyp] OR Review[ptyp] OR Case Reports[ptyp]

OR Comment[ptyp] OR Corrected and Republished Article[ptyp])

AND ("1999"[PDAT] : "2009"[PDAT])

The different types of PubMed query elements are broken down as follows:

[mesh]: Medical Subject Heading (MeSH). At least one (usually several) are manually applied to every article indexed in PubMed by National Library of Medicine (NLM) cataloguers, based on content.

[Title/Abstract]: Free text present in article title or abstract.

[ptyp]: Publication type, including format (journal article v. conference proceedings) and genre (commentary v. meta-analysis).

[lang]: Language of article. Does not affect institution or nation of origin.

[pdat]: Publication date. Does not affect date article was indexed in PubMed.

Each query element we used is broken down as follows, along with a reason for its inclusion. Terms that were used as exclusionary criteria (i.e. preceded by the Boolean operator “NOT”) are italicized and described accordingly.

Humans[mesh]: This is applied to all studies with human subjects.

Magnetic resonance imaging[mesh]: This is applied to all studies using or discussing the use of structural or functional magnetic resonance imaging.

fmri[Title/Abstract]: This, along with all other permutations of “functional magnetic resonance imaging,” is used to identify fMRI studies based on text searching, as there is no MeSH term to distinguish them from other MRI articles.

*Magnetic resonance imaging/ethics[mesh]:* This term was used as exclusionary criteria because it identifies only articles which discuss the ethics of MRI research – none of which, upon review, are original lab research.

Journal article[ptyp]: This is applied to all full journal articles to distinguish them from conference proceedings and other NLM-indexed documents.

English[lang]: Only English-language documents.

*Editorial[ptyp]:* This, along with all other document genre types, is used to exclude all but original lab research studies, which do not have a specified publication type in PubMed.

1999[pdat]:2009[pdat]: This identifies only articles published between 1999 and 2009.

We employed a system developed for comparing the relative precision (accuracy) and recall (exhaustiveness) of two PubMed result sets in order to maximize the return from the query strategy [1]. We also used this system to clean article metadata inherited from an earlier project using the NCBI batch citation matcher, available as part of the NLM Entrez utilities.

Appendix C. Coding Scheme

| **Concerns** | **Key** | **Description** |
| --- | --- | --- |
| Working with vulnerable groups | 1 | Any explicit mention of considerations that arise from recruiting or working with vulnerable groups, including children, patients in minimal or altered states of consciousness (MCS), people with dementia or compromised cognitive or reasoning abilities. |
| Informed consent | 2 | Any mention of informed consent, or discussion about participant understanding. |
| Incidental findings | 3 | Any mention of incidental findings (how about other terms e.g., accidental … unexpected?) |
| Technical limitations, interpretation and validity of results | 4 | Any article that explicitly addressed limitations, validity, or interpretation of technical aspects in any context. |
| Risks, safety and costs | 5 | Any explicit mention of risks or safety concerns in conducting fMRI research, clinical, including psychological risks. |
| Confidentiality, privacy, and mind-reading | 6 | Any discussion of neuroimaging technologies allowing unwanted access by some other entity to the contents or implications of the contents of one’s mind or brain. |
| Clinical treatment and surgery | 7 | Any application of fMRI relevant to surgery or clinical treatment, including neurofeedback and treatment efficacy. |
| Diagnostic and predictive potential | 8 | Exclusively clinical/psychological, e.g., diagnosis of MCS, predicting future disease or disorder or antisocial personality traits, including enhancement and treatment debates. |
| Forensic, security and military use, neuroeducation | 9 | Law and legal concepts. |
| Commercial use | 10 | fMRI in commercial context (advertising/marketing) and technology for sale. |
| Public communication and education | 11 | Public communication of science (including in educational context), but not advertising. |
| "Meta-neuroethics" - critique or review | 12 | Neuroethics or its definition/direction, including reviews of context-crossing neuroethics issues, also including philosophy of science. |
| Neural correlates of ethically relevant concepts | 13 | Broadly, the “neuroscience of ethics”, i.e., any trial or concept explicitly linked to an ethical or moral judgement or question (morality, empathy, emotions, decision-making, free will and responsibility. |

**Appendix D. CiteSpace Methodology**

We extracted Parts-of-Speech (POS) tags from the article titles, abstracts, ISI descriptors, and identifiers, identifying noun phrases of between 2 and 4 words. We generated a document co-citation network (DCN) by creating nodes for the top cited references based on linear threshold interpolation – 77 articles from the original query output (Interval beginning point c=2,cc=3,ccv=15; midpoint c=3,cc=3,ccv=20; end c=3,cc=3,ccv=20). Links were cosine-weighted within one-year data slices, each of which was individually pruned in order to ensure proportional representation for each year’s publication data within the merged network.

Machine-derived cluster labels were extracted from article abstracts and ranked by three different algorithms: tf*idf (Term frequency
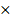
 Inverse document frequency), log-likelihood ratio (LLR) tests, and mutual information [2]. Spectral clustering was used to arrange and visualize this information.

**Appendix E. Scopus Query Construction**

The first of the three queries used to retrieve ethics-content articles, by their source titles are:

*ethic*, poli*, philosophy, law, OR

ISSN matching "00930334" (Hastings Center Report), "0020174X" (Inquiry), "09515089" (Philosophical Psychology), "01638548" (Human Studies), "13557858" (Ethnicity and Health), "10158146" (Genetic Counseling), "08989621" (Accountability in Research), "10790969" (Health and Human Rights)

The query elements are:

“*ethic*” was used to identify any and all permutations of the morpheme “ethic,” include e.g. “neuroethics” and “ethical.”

“poli*” was used to identify journals of policy, or perhaps political theory.

“philosophy” should be self-explanatory, though it is worth noting that the wildcard “philosoph*” was *not* used to exclude journals including the “philosophical transactions” naming convention, many of which were focused on the natural sciences rather than ethics or the humanities.

“law” was used to identify law journals which may contain legal reviews which address the topic of fMRI. This was found to be a more salient naming convention than “legal,” at least for source titles.

*“moral”* was *not* used here or elsewhere as it returned studies of moral correlates in the brain exclusively rather than inquiries into ethical research.

The second of the queries used to retrieve ethics-related articles, by text in their titles and abstracts, are:

*ethic*, justice, responsibil*, stigmati*, personhood

“informed consent” [title]

“*ethic*” was used to identify any and all permutations of the morpheme “ethic,” include e.g. “neuroethics” and “ethical.” This resulted in the greatest number of “moral correlate” studies, but we found no better way of disambiguating the two at the query level because their vocabularies are so similar.

“justice” was one of only two law-related terms which was found to be sufficiently specific that it did not select unrelated boilerplate mentions of “law” or “legality.”

“responsibil*” was the other of two law-related terms which did not retrieve many false hits. Note that the wildcard term is designed to select “responsibility” but not “responsible,” as the latter is common language in describing any cause-and-effect relationship.

“stigmati*” was used to retrieve discussions of the social stigmatization of individuals whose fMRI results had indicated as being non-normal.

“personhood” was used to retrieve discussions of subjects’ individualism being limited by their brain chemistry, as has been called “neuroessentialist.”

“informed consent” was used *only* as a search string for article titles to retrieve articles which were actually *about* the process of granting informed consent. A small but substantial portion of unrelated articles included their boilerplate mention of informed consent in the abstract, rendering this term too broad for the purpose of searching abstracts.

The third search strategy used, the bioethics[sb] PubMed subset, was designed by the Kennedy Institute of Ethics, Georgetown University. It is available in full from: <http://www.nlm.nih.gov/bsd/pubmed_subsets/bioethics_strategy.html>.

**References**

1. Garnett A, Piwowar H, Rasmussen E, Illes J (2010) Expediting medical literature coding with query-building. In the Proceedings of the 73rd Annual Meeting of The American Society for Information Science and Technology.

2. Chen C, Ibekwe-SanJuan F, Hou J (2010) The structure and dynamics of co-citation clusters: A multiple-perspective co-citation analysis. Journal of the American Society for Information Science and Technology 61(7): 1386-1409.
